# Transdisciplinary allied health assessment for patients with stroke: a pre-/post- mixed methods study protocol

**DOI:** 10.1186/s12913-022-08926-y

**Published:** 2022-12-24

**Authors:** Aleysha K. Martin, Theresa L. Green, Alexandra L. McCarthy, P. Marcin Sowa, E-Liisa Laakso

**Affiliations:** 1 Faculty of Medicine, Mater Research Institute – University of Queensland, QLD, Brisbane, Australia; 2 School of Nursing, Midwifery and Social Work, The University of Queensland, QLD, Brisbane, Australia; 3 School of Nursing, Midwifery and Social Work, Mater Research Institute – University of Queensland, QLD, Brisbane, Australia; 4 Centre for the Business and Economics of Health, The University of Queensland, QLD, Brisbane, Australia; 5 Allied Health, Mater Research Institute – University of Queensland, QLD, Brisbane, Australia

**Keywords:** Transdisciplinary, Assessment, Allied health, Stroke, Acute stroke unit, Health services research, Protocol, Mixed methods

## Abstract

**Background:**

Transdisciplinary approaches can streamline processes and build workforce capacity by blurring traditional responsibilities and integrating aspects of care. Emerging evidence shows transdisciplinary approaches can improve time-efficiency, quality of care and cost-effectiveness across various healthcare settings, however no empirical study is based on an acute stroke unit.

**Methods:**

The SPIRIT checklist was used to guide the content of the research protocol. The study is a pragmatic pre−/post- mixed methods four-phase study with a 3-month follow up, based at the Mater Hospital Brisbane. Participants experiencing stroke symptoms will be recruited as they are admitted to the acute stroke unit. Patients presenting with mild stroke symptoms or Transient Ischaemic Attack will be allocated to Phase 1 (baseline) or Phase 2 (implementation), while patients presenting with moderate to severe stroke symptoms will be allocated to Phase 3 (baseline) or Phase 4 (implementation). Participants in baseline Phases 1 and 3 will receive standard allied health assessment, while participants in implementation Phases 2 and 4 will receive the novel transdisciplinary assessment. For the primary aim, allied health professionals will time their assessments to evaluate time taken to administer a novel transdisciplinary assessment, compared to usual discipline-specific assessments. Non-inferiority of the novel transdisciplinary assessment will also be explored in terms of patient safety, compliance to national standards, use of the assessment, and stakeholder perceptions. A retrospective medical record audit, staff focus group, patient/staff surveys, and patient phone interviews at 3-months will be completed. Quantitative results will be estimated using general linear and logistic regression models in Stata 15.1. Qualitative results will be analysed using frequency counts and NVivo software. An economic evaluation will be performed using three scopes including the allied health assessment, hospital admission, and patient outcomes at 3-months.

**Discussion:**

When designing the study, pragmatic factors related to staff willingness to be involved, patient safety, and existing clinical pathways/processes were considered. To address those factors, a co-design approach was taken, resulting in staff buy-in, clinically relevant outcome measures, and the pre−/post- four-phase study design.

**Trial registration:**

Australian New Zealand Clinical Trials Registry (ANZCTR), ACTRN12621000380897. Registered 06 April 2021 - retrospectively registered, https://www.anzctr.org.au/Trial/Registration/TrialReview.aspx?id=381339&isReview=true

**Supplementary Information:**

The online version contains supplementary material available at 10.1186/s12913-022-08926-y.

## Background

Allied health professionals (AHPs) are central to the provision of quality stroke services. The Australian stroke guidelines state that all people experiencing stroke should receive physiotherapist assessment within 24–48 hours of admission to hospital, and assessment by occupational therapists and speech pathologists within 48 hours of admission [1]. While each AHP has a clearly defined role, overlapping skills and knowledge result in some assessment tasks and questions being administered repeatedly by different clinicians [2–4]. At the Mater Hospital Brisbane (MHB) Acute Stroke Unit (ASU), the physiotherapist, occupational therapist, and speech pathologist assess patients presenting with stroke separately. However, these discipline-specific assessments overlap, for example the physiotherapist and occupational therapist both assess upper limb function and gather information on previous level of function and the home environment. Such duplication across allied health assessments is unnecessary, inefficient, and AHP time could be better utilised.

A transdisciplinary approach to allied health stroke assessment could streamline AHP services, which is pertinent given the increasing consumer demand for stroke care. In Australia, the number of new stroke diagnoses per year is predicted to grow from 27,000 in 2020 to exceed 50,000 by 2050 [5]. Transdisciplinary approaches are defined by their potential to build workforce capacity by blurring lines of traditional responsibilities and integrating aspects of care (such as assessment) to enable a single team member to deliver the occasion of service [6–9]. There is emerging evidence that demonstrates, when compared to usual care, hospital-based allied health transdisciplinary teams improve AHP time-efficiency, quality of care, and are cost-effective [3, 10, 11]. No negative or harmful outcomes were reported [3, 10, 11]. When pilot testing the allied health transdisciplinary assessment to be used in this study, the authors demonstrated a mean time saving of 103 minutes when the transdisciplinary assessment was used (42 minutes), compared to usual single-dicipline assessments (145 minutes) [12]. A time saving of 103 minutes could be clinically significant. For example, AHPs would have more time available to commence rehabilitation earlier in the patient episode of care, thereby aligning the service more closely with national stroke guidelines [1]. The pilot testing served as proof of concept for the study proposed in this paper. To the author’s knowledge, no other empirical studies have evaluated transdisciplinary approaches on ASUs. Due to the low volume of empirical evidence pertaining to transdisciplinary approaches on ASUs, alongside the potential for transdisciplinary approaches to streamline stroke services, empirical research is warranted in this field.

## Methods/design

The SPIRIT checklist was used to guide the content of the protocol (see Additional file 1).

### Study aim

The primary aim of this research is to understand the benefits (or otherwise) of implementing a novel Transdisciplinary Initial Neurological Screening Assessment (TINSA) on the MHB ASU. We hypothesise that compared to the existing discipline-specific allied health stroke assessments, utilising the TINSA will reduce allied health assessment time by at least 20 minutes during the initial occasion of service, without reducing quality of care. Secondary aims are to evaluate use of AHP time across the participant hospital admission, quality of care provided by AHPs in terms of patient safety and compliance to national stroke guidelines, clinical utility of the novel TINSA, economic implications of implementing the novel TINSA, patient and staff satisfaction, staff interprofessional trust, and staff confidence to complete and share transdisciplinary tasks.

### Study setting

This is a single-centre metropolitan clinical study, based at the MHB ASU. The ASU is a dedicated 7-bed unit within the Mater Centre for Neurosciences, which is a mixed publicly and privately funded hospital ward. The ASU is serviced by neurologists, nurses, occupational therapists, physiotherapists, speech pathologists, social workers, dieticians, and allied health assistants.

### Study design

**Fig. 1:**
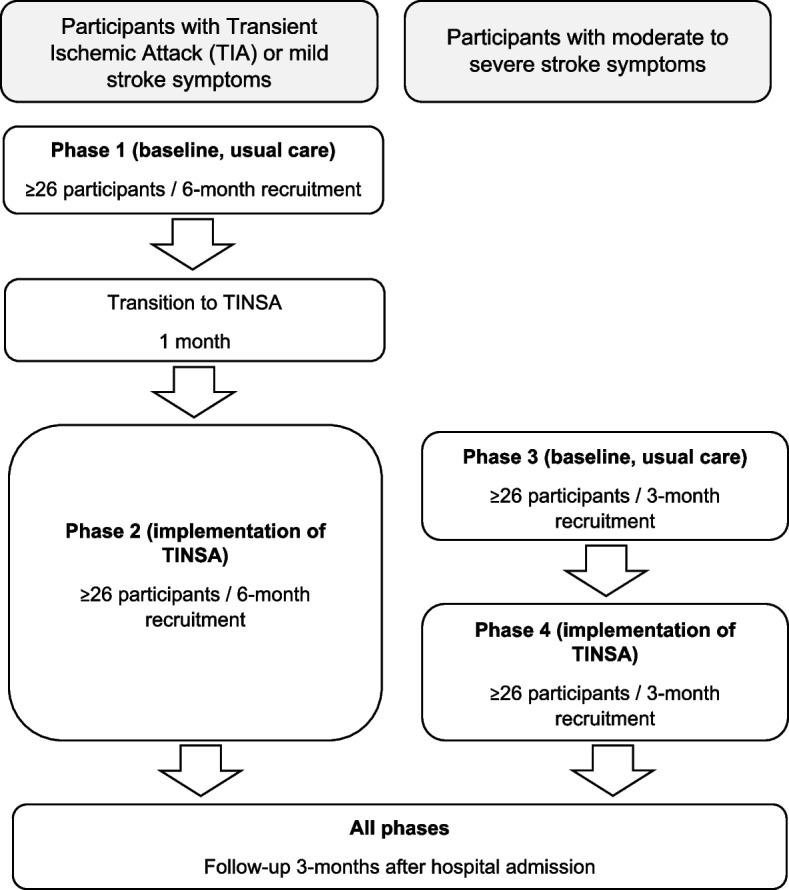
Transdisciplinary study design

The clinical study of the TINSA is a pragmatic pre−/post four-phase mixed methods study with non-randomised comparative groups, a convergent parallel design, and longitudinal follow-up at 3-months after hospital admission. The main quantitative element of the study incorporates descriptive and cross-sectional analytic components and measures designed to evaluate and compare four periods of time during which existing and new transdisciplinary stroke assessments are evaluated (see Fig. 1). Only the allied health stroke assessment component of the patient admission to the MHB ASU will be evaluated, and subsequent allied health input will continue as per usual care. Phase 1 (baseline, usual care) and Phase 2 (implementation of TINSA) will recruit participants experiencing symptoms of mild stroke or Transient Ischaemic Attack (TIA) and evaluate usual assessment compared to the novel TINSA (respectively). Between Phases 1 and 2, a 1-month transition phase will take place to allow staff to become familiar with the novel TINSA, complete competency training, and practice the new clinical process. Phase 3 (baseline, usual care) and Phase 4 (implementation of TINSA) will recruit participants experiencing moderate to severe stroke symptoms to evaluate usual assessment compared to the novel TINSA (respectively). Phases 3 and 4 will occur in parallel with Phase 2 and be time-limited (3 months/phase) due to time constraints on the ASU. Due to the nature of the clinical processes and staffing on the MHB ASU, neither randomisation nor blinding are feasible.

### Participants

Potential study participants will include eligible patients and staff working in the ASU.

#### Inclusion criteria

In Phases 1 and 2, patients who are admitted to the MHB ASU with a possible or confirmed diagnosis of mild stroke or TIA are eligible to participate in the study. Mild stroke or TIA are defined as presentations of mild or resolved neurological symptoms including limb strength above grade 3/5; without visuospatial or sensorimotor neglect; able to communicate verbally; able to follow a one-stage command. From the eligible participants, only those who score ≥ 19/30 on the Montreal Cognitive Assessment (MoCA) (participants with no significant cognitive deficits [13]) will be asked to complete the patient satisfaction survey and follow-up phone call at 3-months after admission.

In Phases 3 and 4, patients who are admitted to the MHB ASU with a possible or confirmed diagnosis of moderate to severe stroke are eligible to participate in the study. Moderate to severe neurological symptoms include limb strength below grade 3/5; visuospatial and/or sensorimotor neglect; aphasia; cognitive impairment. All eligible participants (and/or an authorised representative) will be asked to participate in a follow-up phone call 3-months after admission.

All consenting staff who work on the MHB ASU during the study period will be eligible for inclusion in the study.

#### Exclusion criteria

In all phases, patients will be excluded from the study when admitted to the MHB ASU outside of business hours (i.e., outside of 0800 h – 1630 h weekdays, weekends, or public holidays) when allied health services are not fully staffed; when allied health assessment has already been completed during the admission; if a third-party professional interpreter is unavailable for patients from non-English speaking backgrounds; and/or if the patient has been included in the study previously.

### Processes

#### Recruitment and consent

**Fig. 2:**
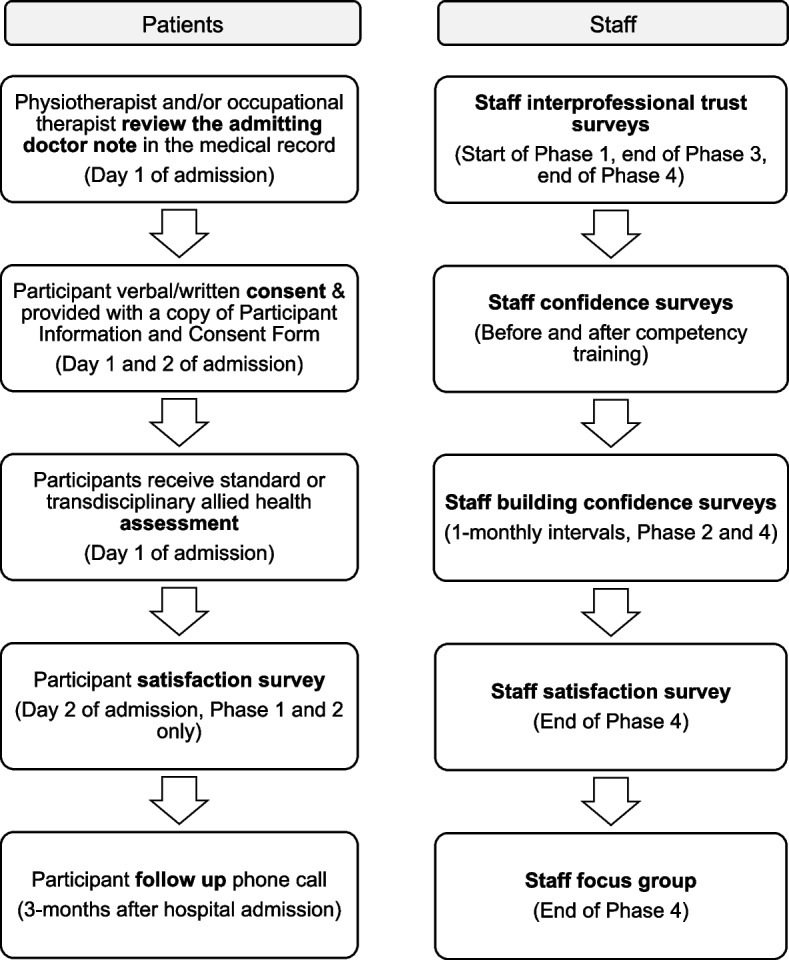
Recruitment timeline for patients and staff

The study procedure for participants and staff is outlined in Fig. 2. Across all Phases, on day 1 of admission to the MHB ASU, patient participants will be recruited using convenience sampling (i.e., as they are admitted). To identify participants who meet the eligibility criteria, the physiotherapists and/or occupational therapists will review the admitting doctor note in the patient medical record. Participants will be provided with a Participant Information and Consent Form and will provide written consent if agreeing to take part in the study (see Additional file 2). Where it is not possible to obtain written consent due to quick discharge from hospital (i.e., before day 2 on the acute stroke unit when consent is obtained), verbal consent will be obtained via phone call, as approved by the ethics committee. On day 2 of admission, eligible patient participants will be asked to partake in a satisfaction survey. At 3-months after admission to hospital eligible participants (and/or authorised representatives) will receive a follow-up phone call to measure functional outcomes and quality of life.

Staff working on the MHB ASU will be provided with a Healthcare Provider Information and Consent Form before agreeing and completing the satisfaction and interprofessional trust surveys (see Additional file 3). The surveys will remain open for 5 business days and returned to a secure slot box or submitted online. Staff will be invited to participate in a focus group at the end of Phase 4. The ASU occupational therapists, physiotherapists, speech pathologists and social workers will be invited to complete a series of online staff confidence surveys before/after completing competency training and at monthly intervals during the implementation Phases 2 and 4. Patients and staff may decline to consent or withdraw their consent to participate at any point and for any reason without prejudice. Upon withdrawal no further data will be collected.

#### Baseline phases 1 and 3

For statistical purposes, the baseline data will be gleaned from Phases 1 and 3 where discipline-specific allied health stroke assessments are utilised within 48 hours for patients admitting to the MHB ASU (i.e., the existing physiotherapy, occupational therapy, and speech pathology stroke assessment).

#### Implementation phases 2 and 4

The novel TINSA (see Additional file 4) replaces discipline-specific allied health stroke assessments and will be utilised within 48 hours of admission to the MHB ASU in Phases 2 and 4. The novel TINSA has been developed in full collaboration with the health professionals on the MHB ASU, including occupational therapy, physiotherapy, speech pathology, social work, nursing staff, and the stroke clinical nurse consultant. The TINSA includes sections on social history, mood, previous level of function, home environment, communication, swallow, respiratory function, vision, cognition, upper limb function, lower limb function, and mobility. Due to the content and clinical skills required, the TINSA can be administered by trained physiotherapists or occupational therapists. The novel TINSA was approved and published in the hospital Policy and Procedure Library prior to use in the study.

Alongside the TINSA, a competency training package has been developed to foster new skill attainment, standardise assessment administration, and support safe implementation of the novel TINSA. The training package consists of four components: 1) a comprehensive manual, 2) quick reference guide, 3) nine eLearning Modules, and 4) a competency assessment. The nine eLearning Modules and competency assessment are mandatory components of the training package, providing targeted learning on topics which were identified as being beyond the usual scope of practice for a new graduate and/or experienced physiotherapists or occupational therapists. Martin and colleagues describe the development of the TINSA and training package in more detail [12].

### Outcome measures

A complete list of outcome measures is provided in Table 1.

**Table 1:** List of outcome measures

Outcome	Measures
Time-efficiency	• Total time of allied health stroke assessments • Time from admission to enactment of social work referral • Time from admission to commencement of allied health intervention/rehabilitation • Occasions of face-to-face service with AHPs (occupational therapy, physiotherapy, speech pathology, social work) • Number of referrals to AHPs (occupational therapy, physiotherapy, speech pathology, social work) where no intervention or further assessment is indicated • Number of patients who received allied health assessment but had a negative stroke diagnosis on MRI • Time from admission to discharge from allied health (occupational therapy, physiotherapy, speech pathology) • Length of hospital stay
Cost savings	• Economic evaluation to determine difference in utilisation of hospital resources, such as bed space and AHP time
Quality of care (patient safety)	• Total number of adverse events during the hospital admission that may be related to allied health input (falls, extension of stroke, aspiration pneumonia, pressure injuries) • Missed allied health referrals (other than those due to a change in condition) • Failed discharge, defined as unplanned readmission within 30 days not related to a new episode of care
Quality of care (compliance to national stroke guidelines)	• Time from admission to allied health assessment (occupational therapy, physiotherapy, speech pathology) • Time from admission to commencement of allied health intervention (occupational therapy, physiotherapy, speech pathology) • Time from admission to mobility assessment • Time from admission to communication assessment
Utility of the novel TINSA	• Number of sections attempted/completed on the novel TINSA • Number of “stop and refer” prompts used on the novel TINSA • Number of allied health referrals identified from the novel TINSA
Patient and staff satisfaction	• Patient satisfaction survey • Staff satisfaction survey • Staff focus group
Staff trust and confidence	• Staff interprofessional trust surveys • Staff confidence surveys (pre/post competency training) • Staff building confidence surveys (monthly intervals)
Patient outcomes at 3-months from hospital admission	• EQ-5D-3L as a measure of quality of life • Modified Rankin Scale (MRS) as a measure of disability • Health event questions pertaining to community falls, hospital readmissions, help from family/friends, formal support services.

#### Time of allied health stroke assessments (primary outcome)

Time taken (minutes) by the AHP to complete the initial stroke assessment(s) will be recorded by each AHP involved in the study including the occupational therapist, physiotherapist, and speech pathologist. The AHPs will record “time in” and “time out” when completing a discipline-specific stroke assessment during baseline Phases 1 and 3, or when completing the TINSA during implementation Phases 2 and 4. Total assessment time includes: 1) time taken to read the medical record, 2) time taken to administer the initial patient assessment (excluding interruptions), and 3) time taken to document assessment results in the medical record.

#### Retrospective medical record audit

A retrospective audit of patient medical records will collect data relating to time-efficiency (e.g., occasions of service), patient safety and adverse outcomes (e.g., number of patient falls), AHP compliance to national stroke guidelines (e.g., assessment completed within 48 hours of admission), and utility of the TINSA (e.g., number of sections completed). The audit of each participant’s medical record will occur at least 2 weeks after patient discharge, to allow sufficient time for the paper medical records to be scanned onto the hospital’s electronic record system (Verdi).

#### 3-month follow up

To assess intermediate-term health outcomes, a follow-up phone call will be conducted by the Principal Investigator at 3-months after admission to the MHB ASU. The EQ-5D-3L will be used to assess self-reported quality of life, where overall health states will be compared [14]. To promote participant retention, the Principal Investigator will attempt to make contact on 3 occasions and leave voice messages. The Modified Rankin Scale (MRS) will be used to assess disability/dependence in activities of daily living after a stroke [15]. The standardised tools and a period of 3-months were selected for three reasons: 1) the timeframe is in line with the follow-up timeframes of 90–180 days used by the Australian Stroke Clinical Registry [16]; 2) standardised interviews using the Modified Rankin Scale are recommended at 3-months (90 days) following hospital discharge [17]; and 3) both measures are used by the National Stroke Foundation and the Australian Stroke Clinical Registry (AuSCR) for follow-up purposes in annual reporting [16]. Health-related questions will also be asked to assess the societal perspective of economic implications, focusing on falls in the community, increase in formal support services or informal supports, and readmissions to hospital.

#### Staff and patient satisfaction surveys

The patient satisfaction survey asks participants to recall their experience of allied health assessment the previous day (see Additional file 5). The survey will be completed in written form if the patient remains in hospital, or via phone call if the patient has discharged from hospital. A paper-based staff satisfaction survey will ask MHB ASU staff to reflect on their experience of the TINSA (see Additional file 6). Both satisfaction surveys will utilise open-ended and multiple-choice questions.

#### Staff interprofessional trust survey

Staff interprofessional trust surveys will be completed during the first week of Phase 1 (i.e., the start of the study), the last week of Phase 3 (i.e., before the novel TINSA is used with the moderate to severe stroke population), and the last week of Phase 4 (i.e., the end of the study). Two surveys will be completed: 1) a validated team trust survey with relevance to healthcare [18]; and 2) a fit-for-purpose questions asking specifically about trust in transdisciplinary teams in healthcare settings (see Additional file 7).

#### Staff confidence surveys

Two online staff confidence surveys will be completed by occupational therapists and physiotherapists, one before and one after competency training. The surveys will ask about staff confidence to administer the TINSA. A series of online confidence surveys will be completed in monthly intervals during implementation Phases 2 and 4 by occupational therapists, physiotherapists, speech pathologists and social workers (see Additional file 8). The surveys ask about confidence to administer the TINSA, confidence to share clinical tasks, and confidence that referrals for other professionals are identified and appropriate.

#### Staff focus group

All staff working on the MHB ASU will be invited to join the staff focus group, to understand perspectives, professional impacts, benefits or drawbacks, value, challenges, utility, and sustainability of the TINSA. The staff focus group will be held at the end of Phase 4. The focus group will use open-ended and prompt questions and will be recorded and transcribed. A limit of 8 participants in the focus group is planned. If more staff are interested in being involved in a focus group, more than one group will be offered.

### Statistical analysis

#### Statistical power

The power calculation demonstrates the required participant number per phase to sufficiently power the study to find a minimal clinically important difference in the primary aim. The following time estimates represent the expert opinions of the AHPs working on the MHB ASU. The variation in time taken to complete discipline-specific occupational therapy, physiotherapy and speech pathology initial stroke assessment may range from 80 to 210 minutes total. The smallest clinically important improvement in time taken to allied health stroke assessment would be a decrease of 20 minutes in average total assessment time (i.e., enough time to complete another occasion of service or clinical task). Assuming a similar distribution of patients in each assessment group, a significance level of 0.05, 80% power and a standard deviation of 25 minutes, 26 patients per group will be required to detect a 20-minute difference in total assessment time.

#### Quantitative data analysis

For each participant group, two periods of time to measure standard discipline-specific allied health assessment and the TINSA assessment will be evaluated and compared. Patient characteristics, outcomes and quantitative survey responses will be summarised for each assessment group using frequency and percent for categorical variables, means and standard deviations for approximately normally distributed continuous variables, and medians and interquartile ranges for non-normally distributed continuous variables. Differences between assessment groups will be estimated using the general linear model for continuous outcomes and logistic regression for categorical outcomes. Balance of potential confounders between the assessment groups will be investigated and multivariable adjustment will be employed where there is an apparent confounding effect. Results will be presented as differences (continuous outcomes) and odds ratios (categorical outcomes) with 95% confidence intervals. All analyses will be performed using Stata 15.1 (Stata Corp, College Station, TX) and a *p*-value < 0.05 will be considered statistically significant throughout all inferential analysis.

#### Qualitative data analysis

Open-ended survey responses will be thematically analysed using NVivo Pro software (QSR International). For the semi-closed questions and Likert scales, percentages will be calculated to show how many participants agreed with a pre-defined answer on the survey.

#### Economic evaluation

An economic evaluation will be performed alongside this study. Compared to usual discipline-specific assessment, we seek to determine the change in engagement of hospital resources when the novel TINSA is utilised with patients experiencing stroke. We expect the potential cost-saving to occur at the initial assessment phase of the hospital admission, while other costs incurred during or after the hospital admission will not be affected. The mean cost per patient will be calculated using a decision tree analysis with expected values calculated from observed patient scenarios. To do this, the analysis will take the approach of analysing outcomes in 3 different scopes from the perspective of the health sector (i.e., MHB where the study will take place). Scope 1 will evaluate the initial assessment phase of the hospital admission. Use of resources including AHP time and bed space will be evaluated using AHP assessment times and hospital coded data. Scope 2 will encompass the entire acute hospital admission (inclusive of Scope 1) with multiple measures available from patient medical records and administrative data collection such as occasions of service by AHPs, counts of adverse events (e.g., aspiration pneumonia), and hospital cost per diem (including equipment, consumables, maintenance, and overheads). Scope 3 will be the broadest view, evaluated 3-months after admission to the MHB ASU. In this scope, an extended healthcare perspective will be used, where relevant health costs and healthcare consequences for the patient post-discharge will be considered. For example, counts of adverse events (e.g., number of falls and unplanned readmissions to hospital), quality of life at 3 months measured using the EQ-5D-3L, and increased reliance on informal/formal supports will be compared between groups and (where possible) compared to national medians/means reported by the Australian Clinical Stroke Registry (AuSCR). A one-way sensitivity analysis will be performed to evaluate uncertainty in the cost estimates regarding the TINSA. For example, the costs of AHPs will be examined at the low and high ends of the clinical pay scale for the MHB ASU (e.g., from Health Practitioner Level 3.1 to Level 7.2) [19, 20]; the time of the TINSA assessment will be varied to represent two standard deviations above the mean (i.e., the scenario where costs calculated account for 95% of observations); and length of stay will be varied to represent two standard deviations above/below the mean to capture changes in hospital cost per diem (including equipment, consumables, maintenance, and overheads) for 95% of observations.

### Data management and monitoring

The Principal Investigator (AM) will be responsible for distribution and collection of surveys, medical record audits, completing the 3-month follow-up phone calls, and entering all data into a password-protected Excel worksheet. The Excel worksheet will be stored in a secure file and only the Principal Investigator will know the password. Data will be stored using numbers, where all categorical variables (e.g., discharge destination) will be assigned a numeric code. Once all participant data has been entered into the Excel worksheet and the study has closed, the data column containing the unique participant identifiers will be deleted, leaving only the data of interest for statistical analysis. Participant data will become unidentifiable at that point. Following analysis, the data will be deposited in an appropriate institutional data repository (The University of Queensland eSpace).

During the study period, the MHB ASU team will take on the role of the Data Monitoring and Safety Committee (DMSC). The DMSC will meet each fortnight to review study conduct and patient safety. The pre-specified review, stopping and discontinuation rules include if 1) data are collected, managed and/or stored outside of the planned methods; 2) data is unable to be de-identified; and 3) there is an increase in adverse outcomes including patient falls, missed allied health referrals and/or failed discharges during Phases 2 or 4 (TINSA implementation) when compared to Phases 1 or 3 (baseline). If adverse outcomes are deemed to have occurred because of the study, use of the TINSA will cease, patients will receive usual care, and any serious adverse events will be reported through the hospital procedure channels and to the institutional Human Research Ethics Committee (HREC).

### Dissemination strategy

The study results will be released to clinical staff and managers working on MHB ASU via written communication and in-services; participants who have indicated interest in receiving results via written communication; and publicly via a peer-reviewed publication. All authors will contribute to results and manuscript preparation, and it is estimated that results will be disseminated in 2023 to 2024.

## Discussion

Originality of the study was considered and influenced study design. To the author’s knowledge, this is the first empirical study to evaluate a transdisciplinary approach on an ASU. Due to the novel setting, it was important to acknowledge the potential for an efficiency-quality trade-off, even though transdisciplinary approaches in other healthcare settings (such as medical wards or community settings) have been shown to improve time-efficiency and achieve improved or equivalent quality of care [2, 3, 11]. Therefore, the study has a dual focus on time-efficiency and quality of care, recognising that earlier and faster assessment should not be at the expense of the quality of AHP assessment, patient safety, or patient outcomes. Accordingly, a range of time-efficiency and quality outcomes are included in the study.

Pragmatic factors related to capacity and willingness of AHPs to be involved in the research, patient safety when receiving the transdisciplinary assessment, and integration of the transdisciplinary approach with existing clinical pathways were also considered when designing the clinical study. To address these factors, a co-design approach between researchers and clinicians was taken. Firstly, the co-design approach secured clinical staff buy-in and involvement in the research. For example, the AHPs contributed to the development of the novel TINSA and study design, increasing investment and willingness to participate in data collection by recording their assessment time.

Secondly, the co-design approach resulted in a feasible and robust study design that mitigated potential implementation barriers, including concerns for patient safety. AHPs were concerned for the scenario where a patient with moderate to severe stroke is being assessed by a clinician who is working outside of their usual scope of practice (e.g., an occupational therapist completing the initial mobility assessment). To mitigate the concerns, the four-phase study design was selected as it allows for staged implementation of the novel TINSA. In other words, initially the TINSA will only be used with patients experiencing TIA/mild stroke symptoms. This will provide staff with time and opportunity to practice the TINSA, become comfortable and confident, and gain perspective regarding how the TINSA could be suitable and safe for patients experiencing more severe stroke.

Consulting with staff also ensured clinically meaningful outcome measures and an appropriate study design were selected. As all AHPs need to know what assessment is being completed with patients (i.e., the novel TINSA or discipline-specific assessments), a blinded study was not possible. Furthermore, parallel study groups (as required for a Randomised Controlled Trial) were not practical. Parallel study groups would require two clinical pathways for the assessment and management of stroke patients. Treating one patient group using two clinical pathways was viewed as confusing, would risk inconsistent care and missed assessments, and complicate clinical handover between AHPs. Additionally, study results obtained from parallel groups might not reflect the “real-world” where only one clinical pathway is used. For example, staff satisfaction responses could be skewed, where staff reflect on the experience of navigating two clinical pathways rather than reflecting on the experience of utilising the TINSA. Or time-efficiency results might not be accurate, as AHPs would take extra time to determine which patient is on what clinical pathway.

### Limitations and implications

There is one main limitation of the study. This is a single-centre study, which means the processes undertaken in developing and carrying out this research might not be suitable in other clinical settings without adaptation and consideration of the local context. The novel TINSA has been developed by AHPs (content experts) at the MHB. While the AHPs have clinical experience at MHB as well as external settings, the novel TINSA has been tailored for the MHB ASU. Adaption and consideration of the local context would be required before implementing and evaluating the novel TINSA in other settings. Despite this, the protocol paper is intended to provide a platform and guide future empirical transdisciplinary research.

## Supplementary Information

**Additional file 1.** SPIRIT Checklist. Description. Completed SPIRIT Checklist.

**Additional file 2.** Patient Information and Consent Form. Description. The Patient Information and Consent Form used to obtain patient consent to participate in the study.

**Additional file 3.** Healthcare Information and Consent Form. Description. The Patient Information and Consent Form used to obtain patient consent to participate in the study.

**Additional file 4.** Transdisciplinary Initial Neurological Screening Assessment (TINSA)© 2022 Mater Misericordiae Limited ABN 83906708922. Description. A copy of the novel TINSA developed at the Mater Hospial Brisbane Acute Stroke Unit. The study protocol describes how the novel TINSA is being evaluated, compared to usual allied health assessment.

**Additional file 5.** Patient Satisfaction Survey. Description. Data collection form used to obtain patient satisfaction data.

**Additional file 6.** Staff Satisfaction Survey. Description. Data collection form used to obtain staff satisfaction data.

**Additional file 7.** Interprofessional Staff Trust Survey. Description. The fit-for-purpose data collection form (i.e., not the validated team trust survey) used to obtain interprofessional staff trust data.

**Additional file 8.** Staff Confidence Survey. Description. Data collection form used to obtain staff confidence data over time.

## Data Availability

Not applicable.

## Abbreviations

AHPs: Allied Health Professionals
MHB: Mater Hospital Brisbane
ASU: Acute Stroke Unit
TINSA: Transdisciplinary Initial Neurological Screening Assessment
TIA: Transient Ischaemic Attack
MRS: Modified Rankin Scale
DMSC: Data Monitoring and Safety Committee
HREC: Human Research Ethics Committee
RGO: Research Governance Office
ANZCTR: Australian New Zealand Clinical Trials Registry
