# Supplementary material for: Transdisciplinary allied health assessment for patients with stroke: a pre-/post- mixed methods study protocol
Source: BMC Health Serv Res. 2022 Dec 24;22:1578. doi: 10.1186/s12913-022-08926-y (PMC9789550; doi:10.1186/s12913-022-08926-y)
Supplement: Supplementary file 2 — Additional file 2. Patient Information and Consent Form. Description. The Patient Information and Consent Form used to obtain patient consent to participate in the study. [file 12913_2022_8926_MOESM2_ESM.pdf]

Document Title:	Participant Information and Consent Form
Unit Record No.:	
Surname:	
Given Names:	
DOB:	

F 3 3 6 3

Participant Information Sheet and Consent Form

Interventional Study - *Adult providing own consent*

Mater Hospital Brisbane

**Title**	Transdisciplinary stroke assessment: Can it improve allied health efficiency and care on an acute stroke unit?
**Short Title**	TINS Project
**Ethics Approval Number**	66933
**Coordinating Principal Investigator/ Principal Investigator**	Aleysha Martin
**Associate Investigator(s)**	Liisa Laakso, Alexandra McCarthy, Theresa Green, Marcin Sowa
**Location**	Acute Stroke Unit, Neurosciences Ward, Level 8, Salmon Building, Mater Hospital Brisbane

1 Introduction

You are invited to take part in this research project because you have had a transient ischaemic attack (TIA), a possible stroke or a mild stroke. The research project is testing a new method of allied health assessment. Allied health staff include people like occupational therapists, physiotherapists, speech pathologists and social workers.

This document tells you about the research project. Knowing what is involved will help you decide if you want to take part in the research.

Please read this information carefully. Ask questions about anything that you don't understand, or you want to know more about. Before deciding if you would like to take part, you might want to talk about it with a relative, friend or your doctor.

You will be given a copy of this Participant Information and Consent Form to keep. If you decide you want to take part in the research project, you will be asked to sign the consent section. By signing it you are telling us that you:

- Understand what you have read
- Consent to take part in the research project
- Consent to have the tests and treatment that are described, and
- Consent to the use of your personal and health information as described.

2 What is the purpose of this research?

The aim of the research is to study the benefits and/or barriers of using a different allied health assessment approach in the Mater Acute Stroke Unit. Normally, on the first or second day of your admission to the Stroke Unit, the allied health staff would each come to see you separately to talk to you about your condition and to do initial tests of your physical and mental abilities before prescribing your treatment or follow-up. The new approach called the TINS means that on the first day of your admission to the stroke unit, only one allied health professional would come to see you to do the initial assessment.

In the study, we will compare each approach. After the initial testing, everything else remains the same as usual with your care including the possibility of further assessment. Be assured that the way the allied health staff treat you after the initial assessment will remain the same as usual, and the way the doctors and nurses assess and treat you will not change. You will continue to receive the high quality of care for which the Mater is known.

3 What does participation in this research involve?

After admission to the Mater Acute Stroke Unit, the investigators will read your doctor's admitting notes to determine if you are eligible for this study. If you agree to participate, you will be asked to sign the consent form and you will be given a unique code number where any information collected for the research will be recorded.

As a participant you will have three responsibilities:

1. On day 1 at the Acute Stoke Unit you were assessed by at least one allied health professional. The assessment included questions and tests that anyone with your medical history is routinely asked. The results helped to determine what treatment you might need in hospital and/or at home. Each assessment was timed and took 35 – 60 minutes. The assessment form was placed into your medical notes, as is-usual practice. There is no further change to allied health care after the assessment.
2. On day 2 at the Acute Stroke Unit, you could see allied health professionals for continuing care as has been determined necessary for your treatment. Also, the investigators may ask you to complete a satisfaction survey. The survey will ask for your feedback about the allied health assessment you received the previous day. This survey will take 5-10 minutes to complete.
3. After 3 months from your admission to the Acute Stroke Unit, the principal investigator (Aleysha Martin) may phone you to ask you some questions. The questions will ask you about your walking, self-care, usual activities, pain, anxiety, depression, health state and level of disability. The questions will take 15-20 minutes to answer. Once you have completed the follow up phone call, your participation in the study is finished.

In the study, you also consent for the investigators to review your medical notes after your discharge from the Mater to collect information on the time efficiency, safety and compliance of the assessment process with national stroke standards. This research project has been designed to make sure the researchers interpret the results in a fair and appropriate way to avoid investigators or participants jumping to conclusions.

4 How do I provide consent on behalf of a participant?

As a an authorised representative, you can support a participant by reviewing this information and consent form together. You can discuss and decide if involvement, benefits and risks are acceptable to participate in the research. Under the circumstances that the patient is unable to give consent, you will have authority to sign on behalf of the participant as a substitute decision maker if you are listed as a contact in the patient's admitting record.

5 Other relevant information about the research project

Each month, we estimate at least 16 participants will take part in this research project; and we are aiming for at least 100 patients over the 12-18 month duration of the study. Depending on the results of this study, we may continue using the TINS or expand the study to include people admitting with severe stroke at the Mater Hospital Brisbane. If it proves safe and useful, the TINS may become used at other hospitals with Acute Stroke Units.

6 Do I have to take part in this research project? What if I decide to withdraw?

Participation in any research project is voluntary. If you decide to take part, you will be given this Participant Information and Consent Form to sign and you will be given a copy to keep. If you do not wish to take part, you do not have to. If you decide to take part and later change your mind, you are free to withdraw from the project at any stage. If you decide to withdraw later, please notify a member of the research team. We will discuss any health risks or special requirements linked to withdrawing and ask you to sign a Withdrawal of Consent Form. We will not collect additional personal information from you although personal information already collected will be retained (with your permission) to ensure that the results of the research project can be measured properly and to comply with law. You should be aware that data collected up to the time you withdraw will form part of the research project results. If you do not want this, you must tell the researcher before you join the research project. Your decision whether to take part or not to take part, or to take part and then withdraw, will not affect your routine treatment, your relationship with those treating you or your relationship with Mater Hospital Brisbane.

7 What are the alternatives to participation?

You do not have to take part in this research project to receive treatment at this hospital. Other options are available: standard allied health assessment by physiotherapy, occupational therapy, speech pathology and social work.

8 What are the possible benefits of taking part?

We cannot guarantee or promise that you will receive any benefits from this research however, possible benefits might include: knowing that you are contributing to making health care more efficient; allied health assessment could take less time; different allied health professionals will not repeat the same assessment questions/tasks; and you might experience a quicker discharge from allied health services and/or from hospital. There are no additional costs associated with participating in this research project, nor will you be paid.

9 What are the possible risks and disadvantages of taking part?

Hospital admissions can have unknown risks. You might not experience any, some or all the risks listed below, and they could be mild, moderate or severe. There could be risks that the researchers do not expect or do not know about and that are potentially serious. These are risks that could occur during any hospital admission. If you are worried about them, talk with the Principal Investigator (Aleysha Martin) or your ward doctor.

Risk	How often is it likely to occur?	How severe might it be?	How long might it last?
Missed allied health referral	Rarely	Mild	Temporary
Fall at hospital leading to possible injury	Rarely	Mild to severe	Temporary to permanent
Hospital readmission due to poor allied health discharge plan	Rarely	Mild to severe	Temporary
Disclosure of sensitive personal information	Rarely	Mild to severe	Temporary to permanent

10 Could this research project be stopped unexpectedly?

This research project could be stopped unexpectedly for a variety of reasons, such as:
- The TINS could be associated with a higher number of patient falls;
- The TINS needs further testing.

11 What happens when the research project ends?

You will be provided with the contact details of your principal investigator (listed on this document). When your involvement in the research project ends, you will be able to initiate follow-up or request results using these details. Results will be available on the completion of the study (approximately July 2022). If the study results show benefits of the TINS, this assessment will become available to all people with stroke admitting to the Mater Hospital Brisbane Acute Stroke Unit.

12 What will happen to information about me?

By signing the consent form, you consent to the research staff collecting and using de-identified personal information about you for the research project. Any information obtained in connection with this research project that can identify you will remain confidential. Only the Principal Investigator will be able to access your information. The information will be stored for the duration of the project (estimated until July 2022) and data will be kept as per the Mater Hospital policy. The information that you have provided to us will be recorded using your code number in a password-protected file at the Mater Hospital. Any other information related to your admission will be kept in your medical notes as is usual practice. Your information will only be used for the purpose of this research project and it will only be disclosed with your permission, except as required by law. Your identity will become anonymous at the end of the study, when we will remove any link between your identity and the information you have provided to us.

Your health records and any information obtained during the research project are subject to inspections (for the purpose of verifying the procedures and the data) by the relevant authorities and authorised representative of the institution relevant to this Participation Information Sheet (Mater Health), or as required by law. By signing the Consent Form, you authorise release of, or access to, this confidential information to the relevant study personnel and regulatory authorities as noted above.

It is anticipated that the results of this research project will be published or presented in a variety of forums. In any publication or presentation, information will be provided in such a way that you cannot be identified, except with your permission.

13 Complaints and compensation

If you suffer any injuries or complications as a result of this research project, you should contact the research team as soon as possible and you will be assisted with arranging appropriate medical treatment. If you are eligible for Medicare, you can receive any medical treatment required to treat the injury or complication, free of charge, as a public patient in any Australian public hospital.

For complaints regarding your treatment by members of staff involved in this research (occupational therapists, physiotherapists, etc.) contact the clinical contact person. The complaint will be addressed by the principal investigator and the Mater Health Patient Representative will be contacted as necessary/requested.

14 Who is organising and funding the research?

This research project is being conducted by Aleysha Martin. There are no conflicts of interest, although the research is expected to form part of the PhD studies of the principal investigator (Aleysha Martin).

No member of the research team will receive a personal financial benefit from your involvement in this research project (other than ordinary wages). Mater Health might benefit from this research project if, for example, the project results in time savings in allied health assessment.

You will not benefit financially from your involvement in this research project even if, for example, your personal information (the knowledge acquired from analysis of your personal information) proves to be of commercial value to Mater Services.

15 Who has reviewed the research project?

All research in Australia involving humans is reviewed by an independent group of people called a Human Research Ethics Committee (HREC). The ethical aspects of this research project have been approved by the Mater Misericordiae Limited Human Research Ethics Committee, which is the institution responsible for supervising the standard of care where the research will be carried out.

This project will be carried out according to the *National Statement on Ethical Conduct in Human Research (2007, updated 2018).* This statement has been developed to protect the interests of people who agree to participate in human research studies.

This study has been reviewed and approved by the Mater Misericordiae Ltd Human Research Ethics Committee (EC00332). Should you wish to discuss the study in relation to your rights as a participant, or should you wish to make an independent complaint, you can contact the HREC Liaison Officer or HREC Chairperson, Human Research Ethics Committee, Mater Misericordiae Ltd, Raymond Terrace South Brisbane 4101, or telephone (07) 3163 1585, email: research.ethics@mater.uq.edu.au

16 Further information and who to contact

The person you need to contact will depend on the nature of your query.
If you want any further information concerning this project or if you have any medical problems related to your involvement in the project you can contact the Principal Investigator:

Clinical contact person

Name	Aleysha Martin
Position	Occupational Therapist & Principal Investigator
Telephone	(07) 3163 6000
Email	aleysha.martin@mater.org.au

For matters relating to research at the site at which you are participating, the details of the local site complaints person are:

Complaints contact person

Name	Roxanne Regan
Position	Patient Representative
Telephone	(07) 3163 8303
Email	roxanne.regan@mater.org.au

Consent Form

**Title**	Transdisciplinary stroke assessment: Can it improve allied health efficiency and care on an acute stroke unit?
**Short Title**	TINS Project
**Ethics Approval Number**	66933
**Coordinating Principal Investigator**	Aleysha Martin
**Associate Investigator(s)**	Liisa Laakso, Alexandra McCarthy, Theresa Green, Marcin Sowa
**Location**	Acute Stroke Unit, Neurosciences Ward, Level 8, Salmon Building, Mater Hospital Brisbane

Note: All parties signing the consent section must date their own signature.

Declaration by Participant

I have read the Participant Information Sheet, or someone has read it to me in a language that I understand.

I understand the purposes, procedures and risks of the research described in the project.

I give permission for my medical information kept at Mater Hospital Brisbane concerning my disease and treatment to be used for the purposes of this project. I understand that such information will remain confidential.

I have had an opportunity to ask questions and I am satisfied with the answers I have received.

I freely agree to participate in this research project as described and understand that I am free to withdraw at any time during the study without affecting my future healthcare.

I understand that I will be given a signed copy of this document to keep.

Name of Participant (please print) ______________________________

Signature ______________________________ Date ____________________

Declaration by Principal Researcher

I have given a verbal explanation of the research project, its procedures and risks and I believe that the participant has understood that explanation.

Name of Researcher (please print) ______________________________

Signature ______________________________ Date ____________________

mater research

F 3 3 6 3

Withdrawal of Participation Form

**Title**	Transdisciplinary stroke assessment: Can it improve allied health efficiency and care on an acute stroke unit?
**Short Title**	TINS Project
**Ethics Approval Number**	66933
**Coordinating Principal Investigator**	Aleysha Martin
**Associate Investigator(s)**	Liisa Laakso, Alexandra McCarthy, Theresa Green, Marcin Sowa
**Location**	Acute Stroke Unit, Neurosciences Ward, Level 8, Salmon Building, Mater Hospital Brisbane

Note: All parties signing the consent section must date their own signature.

Declaration by Participant

I wish to withdraw from participation in the above research project and understand that such withdrawal will not affect my routine treatment, my relationship with those treating me or my relationship with Mater Health.

Name of Participant (please print) ______________________________

Signature ______________________________ Date ______________________

In the event that the participant's decision to withdraw is communicated verbally, the Researcher must provide a description of the circumstances:

Declaration by Researcher

I have given a verbal explanation of the implications of withdrawal from the research project and I believe that the participant has understood that explanation.

Name of Researcher (please print) ______________________________

Signature ______________________________ Date ______________________
