# Supplementary material for: Transdisciplinary allied health assessment for patients with stroke: a pre-/post- mixed methods study protocol
Source: BMC Health Serv Res. 2022 Dec 24;22:1578. doi: 10.1186/s12913-022-08926-y (PMC9789550; doi:10.1186/s12913-022-08926-y)
Supplement: Supplementary file 3 — Additional file 3. Healthcare Information and Consent Form. Description. The Patient Information and Consent Form used to obtain patient consent to participate in the study. [file 12913_2022_8926_MOESM3_ESM.pdf]

Healthcare Provider Information Sheet and Consent Form

Interventional Study - *Adult providing own consent*

Mater Hospital Brisbane

**Title**	Transdisciplinary stroke assessment: Can it improve allied health efficiency and care on an acute stroke unit?
**Short Title**	TINS Project
**Ethics Approval Number**	66933
**Coordinating Principal Investigator/ Principal Investigator**	Aleysha Martin
**Associate Investigator(s)**	Liisa Laakso, Alexandra McCarthy, Theresa Green, Marcin Sowa
**Location**	Acute Stroke Unit, Neurosciences Ward, Level 8, Salmon Building, Mater Hospital Brisbane<br>Acute Medical Unit, Mater Hospital Brisbane

1 Introduction

You are invited to take part in this research project because you are an allied health, nursing or medical staff member who provides care for patients with transient ischaemic attack (TIA), a possible stroke, a mild stroke, or general medical condition. The research project is testing a new approach for allied health assessment. Allied health professionals include people like occupational therapists, physiotherapists, speech pathologists and social workers. The new approach we are testing is called a transdisciplinary model of assessment (currently known as multidisciplinary on the Acute Medical Unit).

This document tells you about the research project. It explains the tests and treatments involved. Knowing what is involved will help you decide if you want to take part in the research. Please read this information carefully. Ask questions about anything that you don't understand, or you want to know more about. Before deciding whether or not to take part, you might want to talk about it with a relative, friend or a colleague. You may also take this form away with you.

Participation in this research is voluntary. If you don't wish to take part, you don't have to. If you decide you want to take part in the research project, you will be asked to sign the consent section. By signing it you are telling us that you:

- Understand what you have read;
- Consent to take part in the research project;
- Consent to complete the surveys that are described;
- Consent to the use the answers/information from your surveys as described.

2 What is the purpose of this research?

The aim of the study is to evaluate the effects of using transdisciplinary models of assessment in the Mater Acute Stroke Unit (ASU) and Mater Acute Medical Unit (AMU). The transdisciplinary assessments may result in more efficient allied health assessment. This research may contribute to workforce redesign to ensure sustainability of future healthcare services at the Mater and other hospitals.
There is emerging evidence that a transdisciplinary approach to patient care may result in reduced duplication of allied health assessment, increased allied health efficiency, reduced occasions of allied health service, reduced patient length of stay, and improved patient and staff satisfaction. We aim to test if a transdisciplinary model of assessment is an effective approach to assess patients presenting with transient ischaemic attack, a possible stroke, a mild stroke, or general medical conditions.

There is also no evidence that explores staff trust and transdisciplinary approaches. We aim to understand staff satisfaction and staff trust.

The results of this research will be used by the principal investigator, Aleysha Martin, as part of a Doctor of Philosophy Degree. This research has been initiated by the principal investigator, Aleysha Martin.

3 What does participation in this research involve?

As a staff member, you will be asked to complete two trust surveys and, if you work on the Acute Stroke Unit, one satisfaction survey. We will ask for surveys to be completed at the start and end of the study. In the first week of the study, you will be asked to complete two trust surveys which may take about 10-15 minutes in total. In the last week of the study, you will be asked to complete the two trust surveys again (which may take 10-15 minutes). If you work on the Acute Stroke Unit, you will also be asked to complete a short staff satisfaction survey in the last week of the last (which may take 5 minutes). The surveys will ask questions about your role on the Acute Stroke Unit/Acute Medical Unit, benefits and challenges of the transdisciplinary assessment, your confidence in the transdisciplinary assessment, and team trust.

The information will be used to understand staff perspectives, trust and satisfaction with the transdisciplinary assessments. The information will be used alongside data collected from consenting patients on the Acute Stroke Unit, with the aim to determine if we improve care by using the transdisciplinary assessment.

4 What are my responsibilities?

Sign the consent form only after you have made up your mind to take part in this clinical study. If you agree to participate in this study, we ask you to complete the surveys as described above. You agree to allow us to use your survey responses. You also agree to comply with the other conditions in this document. If you cannot, or do not wish to accept this responsibility, then we cannot accept you as a participant in the study.

5 Do I have to take part in this research project?

Participation in any research project is voluntary. If you do not wish to take part, you do not have to. If you decide to take part and later change your mind, you are free to withdraw from the project at any stage. If you decide to withdraw at a later date, we will ask you to sign a Withdrawal of Consent Form. Any de-identified information we have collected from you until the time you withdraw will be retained by the researchers for use in the study.

If you decide to take part, you will be given this Healthcare Provider Information and Consent Form to sign and you will be given a copy to keep.

Your decision whether to take part or not to take part, or to take part and then withdraw, will not affect your relationship with the study team or colleagues with the Mater Hospital Brisbane.

6 What are the possible benefits of taking part?

We cannot guarantee or promise that you will receive any benefits from this research however, possible benefits may include:

- Increasing your knowledge and confidence using a transdisciplinary approach;
- An opportunity to help shape future implementation of the transdisciplinary assessments;
- An opportunity to improve efficiency on the Mater Hospital Acute Stroke Unit/Acute Medical Unit.

7 What are the possible risks and disadvantages of taking part?

New approaches to assessment may have unknown risks. However, there are no expected risks or disadvantages to you from your participation in this research.

8 What if I withdraw from this research project?

If you decide to withdraw from the project, please notify a member of the research team before you withdraw. This notice will allow that person or the research supervisor to discuss any health risks or special requirements linked to withdrawing.

If you do withdraw your consent during the research project, the principal investigator and relevant study staff will not collect additional personal information from you although personal information already collected will be retained to ensure that the results of the research project can be measured properly and to comply with law. You should be aware that data collected up to the time you withdraw will form part of the research project results. If you do not want this, you must tell the researcher before you join the research project.

9 Could this research project be stopped early?

This research project may be stopped unexpectedly for a variety of reasons. These may include reasons such as:

- The transdisciplinary assessment being shown not to be effective;
- The transdisciplinary assessment being shown to work and not need further testing.

10 What happens when the research project ends?

You will continue to provide your usual care and workplace duties. The results will be reviewed and discussed with Mater Hospital Acute Stroke Unit/Acute Medical Unit staff to determine if any adjustments to routine care procedures of patients will be required.

You will be provided with the contact details of your principal investigator (listed on this document). When your involvement in the research project ends, you will be able to initiate follow-up using these details. Provided the study results show benefits of the transdisciplinary assessment, it will become available to people with stroke/medical conditions admitting to the Mater Hospital Brisbane Acute Stroke Unit/Acute Medical Unit.

If you are interested in finding out the results of the project, you can request for the results to be shared with you via email, post or phone call. Results will be available on the completion of the study which is forecast to be after December 2021.

11 What will happen to confidential information about me?

By signing the consent form, you consent to the principal investigator and relevant research staff collecting and using personal information about you for the research project. Any information obtained in connection with this research project that can identify you will remain confidential. Only the researchers directly associated with the study will have access to that information. Only the principal investigator will be able to access your information. The information will be stored for the duration of the project (estimated until December 2021) and data will be kept as per the Mater Hospital policy. Survey responses will be typed onto a password-protected Microsoft Word or Excel document, which only the principal investigator can access. Paper surveys will then be shredded. If you agree to be involved in this study you are agreeing to participate in completing the surveys and the data being used to evaluate the transdisciplinary assessment. Your information will only be used for the purpose of this research project and it will only be disclosed with your permission, except as required by law.

To protect your privacy, no information will be published that could identify you as a participant in this study. We will not be able to identify you from your survey responses. We will collect the following information from you:

- Information from two short surveys on staff trust to understand your levels of team trust and the potential implications for a transdisciplinary model of assessment.
- If you work on the Acute Stroke Unit, information from one short survey to seek your perspectives (a staff satisfaction survey) regarding the benefits/drawbacks of a transdisciplinary model of assessment.

12 Who is organising and funding the research?

This research project is being conducted by Aleysha Martin.

No member of the research team will receive a personal financial benefit from your involvement in this research project (other than ordinary wages). Mater Health may benefit from this research project if, for example, the project assists the Mater to develop a transdisciplinary model of assessment that has a reproducible implementation plan.

You will not benefit financially from your involvement in this research project even if, for example, your personal information (your knowledge acquired from analysis of your personal information) proves to be of commercial value to Mater Services.

There are no conflicts of interest, although the research is expected to form part of the PhD studies of the principal investigator (Aleysha Martin).

13 Who has reviewed the research project?

All research in Australia involving humans is reviewed by an independent group of people called a Human Research Ethics Committee (HREC). The ethical aspects of this research project have been approved by the Mater Misercordiae Limited Human Research Ethics Committee, which is the institution responsible for supervising the standard of care where the research will be carried out.

This project will be carried out according to the *National Statement on Ethical Conduct in Human Research (2007, updated 2018).* This statement has been developed to protect the interests of people who agree to participate in human research studies.

This study has been reviewed and approved by the Mater Misericordiae Ltd Human Research Ethics Committee (EC00332). Should you wish to discuss the study in relation to your rights as a participant, or should you wish to make an independent complaint, you may contact the HREC Liaison Officer or HREC Chairperson, Human Research Ethics Committee, Mater Misericordiae Ltd, Raymond Terrace South Brisbane 4101, or telephone (07) 3163 1585, email: research.ethics@mater.uq.edu.au

14 Who do I contact if I have a question or complaint?

The person you may need to contact will depend on the nature of your query. We have included several contacts for you below.

If you want any further information concerning this project or if you have any medical problems which may be related to your involvement in the project you can contact the Principal Investigator:

Clinical contact person

Name	Aleysha Martin
Position	Occupational Therapist & Principal Investigator
Telephone	(07) 3163 6000
Email	aleysha.martin@mater.org.au

Consent Form

**Title**	Transdisciplinary stroke assessment: Can it improve allied health efficiency and care on an acute stroke unit?
**Short Title**	TINS Project
**Ethics Approval Number**	66933
**Coordinating Principal Investigator**	Aleysha Martin
**Associate Investigator(s)**	Liisa Laakso, Alexandra McCarthy, Theresa Green, Marcin Sowa
**Location**	Acute Stroke Unit, Neurosciences Ward, Level 8, Salmon Building, Mater Hospital Brisbane<br>Acute Medical Unit, Mater Hospital Brisbane

Note: All parties signing the consent section must date their own signature.

Declaration by Participant

I have read the Participant Information Sheet or someone has read it to me in a language that I understand.

I understand the purposes, procedures and risks of the research described in the project.

I give permission for the research team to release information to Mater Services concerning the transdisciplinary assessment for the purposes of this project. I understand that such information will remain confidential.

I have had an opportunity to ask questions and I am satisfied with the answers I have received.

I freely agree to participate in this research project as described and understand that I am free to withdraw at any time during the study without affecting my future healthcare.

I understand that I will be given a signed copy of this document to keep.

Name of Participant (please print) ______________________________________

Signature ______________________________ Date ____________________

Declaration by Principal Researcher

I have given a verbal explanation of the research project, its procedures and risks and I believe that the participant has understood that explanation.

Name of Researcher (please print) ______________________________________

Signature ______________________________ Date ____________________

Withdrawal of Participation Form

**Title**	Transdisciplinary stroke assessment: Can it improve allied health efficiency and care on an acute stroke unit?
**Short Title**	TINS Project
**Ethics Approval Number**	66933
**Coordinating Principal Investigator**	Aleysha Martin
**Associate Investigator(s)**	Liisa Laakso, Alexandra McCarthy, Theresa Green, Marcin Sowa
**Location**	Acute Stroke Unit, Neurosciences Ward, Level 8, Salmon Building, Mater Hospital Brisbane<br>Acute Medical Unit, Mater Hospital Brisbane

Note: All parties signing the consent section must date their own signature.

Declaration by Participant

I wish to withdraw from participation in the above research project and understand that such withdrawal will not affect my work or my relationship with other staff, or my relationship with Mater Health.

Name of Participant (please print) ______________________________

Signature ______________________________ Date ____________________

In the event that the participant's decision to withdraw is communicated verbally, the Researcher must provide a description of the circumstances:

Declaration by Researcher

I have given a verbal explanation of the implications of withdrawal from the research project and I believe that the participant has understood that explanation.

Name of Researcher (please print) ______________________________

Signature ______________________________ Date ____________________
