# Supplementary material for: Transdisciplinary allied health assessment for patients with stroke: a pre-/post- mixed methods study protocol
Source: BMC Health Serv Res. 2022 Dec 24;22:1578. doi: 10.1186/s12913-022-08926-y (PMC9789550; doi:10.1186/s12913-022-08926-y)
Supplement: Supplementary file 4 — Additional file 4. Transdisciplinary Initial Neurological Screening Assessment (TINSA)© 2022 Mater Misericordiae Limited ABN 83906708922. Description. A copy of the novel TINSA developed at the Mater Hospial Brisbane Acute Stroke Unit. The study protocol describes how the novel TINSA is being evaluated, compared to usual allied health assessment. [file 12913_2022_8926_MOESM4_ESM.pdf]

Binding margin - do not write. Do not reproduce by photocopying.
All clinical form creation and amendments must be conducted through Health Information.

F5342

mater health

TRANSDISCIPLINARY INITIAL NEUROLOGICAL SCREENING ASSESSMENT (TINSA)

Unit Record No. ______
Surname ______
Given Names ______
DOB ______ Sex ______
AFFIX PATIENT IDENTIFICATION LABEL HERE

Admitting diagnosis/investigations:

History of presenting condition:

Medical history:

Assessment completed with:

Social History

Living arrangement: ☐ Alone ☐ Partner/Family ☐ Other: ......
Carer role: ☐ Provides care ☐ Receives care ☐ Suspected carer stress
Details (who/how much support): ......
Formal services: ☐ Yes ☐ No Details: ......
☐ **Refer to SW** (e.g. lack of support, complex social situation, suspected carer stress, advanced care planning)

Previous Level of Function and Activity Participation

Occupation: ☐ Full-time ☐ Part-time ☐ Retired Details: ......
Education level: ......
Mobility/transfers: ☐ Independent ☐ SBA ☐ Assist ......
Falls in last 12 months: ☐ Yes ☐ No Reason: ......
Eating/diet: ☐ Independent ☐ SBA ☐ Assist (normal/modified; dentition) ......
Toileting/continence: ☐ Independent ☐ SBA ☐ Assist ......
Dressing: ☐ Independent ☐ SBA ☐ Assist ......
Showering: ☐ Independent ☐ SBA ☐ Assist ......
Meal prep: ☐ Independent ☐ SBA ☐ Assist ......
Shopping: ☐ Independent ☐ SBA ☐ Assist ......
Cleaning: ☐ Independent ☐ SBA ☐ Assist ......
Laundry: ☐ Independent ☐ SBA ☐ Assist ......
Gardening: ☐ Independent ☐ SBA ☐ Assist ......
Medication: ☐ Independent ☐ SBA ☐ Assist ......
Finances: ☐ Independent ☐ SBA ☐ Assist ......
Driving: ☐ Yes ☐ No Alternate transport: ......
Leisure/exercise regime: ......

Home Environment

Housing: ☐ House ☐ Unit ☐ Townhouse ☐ Retirement village ☐ RACF ☐ Other: ......
Ownership: ☐ Owner ☐ Rented ☐ Department of Housing ☐ Other: ......
Stairs: ...... Front – rails ascending: ☐ Left ☐ Right ...... Back – rails ascending: ☐ Left ☐ Right
...... Internal – rails ascending: ☐ Left ☐ Right ...... Side – rails ascending: ☐ Left ☐ Right
Ground level rooms: ......
Upstairs rooms: ......
Bathroom: ☐ Shower recess ☐ Bath ☐ Shower over bath ☐ Hob ☐ Rails ☐ HHSH ☐ FSH ☐ Door ☐ Curtain
☐ Equipment: ......
Toilet: ☐ In bathroom ☐ Separate ☐ Rails ☐ Equipment: ......
Other equipment: ...... Personal alarm: ☐ Yes ☐ No
Previous OT home visit: ☐ Yes ☐ No If YES, details: ......

Cognition

Documented/reported history of cognitive impairment? ☐ Yes ☐ No If YES, details: ......
MSQ: ...... **/10** ☐ Day ☐ Date ☐ Month ☐ Year ☐ DOB ☐ Age ☐ Address ☐ Location ☐ PM ☐ PPM
Insight into deficits: ☐ Yes ☐ Partial ☐ No
Comments: ......

TRANSDISCIPLINARY INITIAL NEUROLOGICAL SCREENING ASSESSMENT (TINSA)

05/22
Ver. 3.00
F5342

© Copyright Mater Misericordiae Limited. All rights reserved.

mater health<br>**TRANSDISCIPLINARY INITIAL NEUROLOGICAL SCREENING ASSESSMENT (TINSA)**	Unit Record No. ______<br>Surname ______<br>Given Names ______<br>DOB ______ Sex ______<br>AFFIX PATIENT IDENTIFICATION LABEL HERE

Communication

Hearing aids? ☐ Nil ☐ Left ☐ Right ☐ ALD required Primary language: ....

Has your speech changed recently (e.g. slurred)? ☐ Yes ☐ No

If YES, details: ....

New difficulties thinking of words, reading, writing, understanding what people say? ☐ Yes ☐ No

If YES, details: ....

Have your family/friends noticed any changes with your communication/language? ☐ Yes ☐ No

If YES, details/changes: ....

Informal communication screen

Observations: language in conversation	☐ Level 1 = Normal language<br>☐ Level 2 = Able to speak in sentences but word finding difficulties evident<br>☐ Level 3 = Speaking only in single words or short phrases – can communicate basic needs/wants<br>☐ Level 4 = Very limited or no verbal output – unable to communicate basic needs/wants<br>☐ Level 5 = Nonsensical or no verbal output – unable to communicate basic needs/wants
Naming/word retrieval	*Name the following objects that I point to:*<br>Bed .... Glass/cup .... Elbow .... Light .... Pillow .... Correct: /5
Auditory verbal communication *(yes/no questions)*	*I'm going to ask you some questions, answer yes or no.*<br>1. Is your name Smith?<br>2. Is your name _____? (say patient's last name)<br>3. Are the lights on in this room?<br>4. Is the door/curtain closed?<br>5. Does March come before June? Correct: /5
Speech clarity	☐ Normal<br>☐ Impaired but easy to understand<br>☐ Impaired and difficult to understand<br>Comments: ....
Observations: pragmatics	☐ Reduced eye contact<br>☐ Flat facial expression/voice<br>☐ Poor conversation turn taking/initiation<br>Other: ....

Sheffield Screening Test for Acquired Language Disorders – Receptive skills

1. Verbal comprehension of single words<br>*I'm going to ask you to point to some of the things in the room...*<br>Door .... Light .... Chair .... Ceiling .... Corner	/1
2. Comprehension of sequential command<br>a. point to the window and then to the door /1<br>b. before pointing to the ceiling, touch the chair/bed /1	/2
3. Comprehension of a complex command<br>*Tap the chair/bed twice with a clenched fist, while looking at the ceiling.*	/1

Stop communication assessment HERE and refer to SP if:

☐ NESB

☐ Scores 3–5 in language in conversation ☐ Scores <5/5 naming ☐ Scores <5/5 yes/no questions

☐ Speech clarity "impaired and difficult to understand" ☐ Unable to follow 1 stage command

4. Recognition of differences in meaning between words<br>*I'm going to read you a list of words and I want you to tell me which is the odd one out...*<br>a. chicken, duck, apple, turkey Response: .... /1<br>b. run, drink, walk, sprint Response: .... /1<br>c. small, large, massive, huge Response: .... /1	/3
5. Comprehension of Narrative<br>a. *I'm going to read you a short paragraph and then ask you a question about it.*<br>*"John went to the shop to buy a pen. When he got there he found that he had forgotten his wallet, so he came home and made himself a cup of tea. What should he have taken with him?"* Response: .... /1<br>b. *I'm going to read you another paragraph.*<br>*"Mrs Smith visited several shops. She bought a newspaper, a cauliflower, a stamp and some sausages. What was the second shop she visited?"* Response: .... /1	/2
**Total receptive skills score**	/9

Binding margin - do not write. Do not reproduce by photocopying.
All clinical form creation and amendments must be conducted through Health Information.

© Copyright Mater Misericordiae Limited. All rights reserved.

mater health

TRANSDISCIPLINARY INITIAL NEUROLOGICAL SCREENING ASSESSMENT (TINSA)

Unit Record No. ____________________

Surname ____________________

Given Names ____________________

DOB ____________ Sex ____________

AFFIX PATIENT IDENTIFICATION LABEL HERE

Binding margin - do not write. Do not reproduce by photocopying.
All clinical form creation and amendments must be conducted through Health Information.

Communication *(continued)*

Sheffield Screening Test for Acquired Language Disorders – Expressive skills

6. Word Finding<br>*Tell me the names of three well-known places* (in the client's home town). Response:	/1
7. Abstract word finding<br>*Tell me another word that means the same as:*<br>a. beautiful Response: .... /1<br>b. angry Response: .... /1<br>c. ridiculous Response: .... /1	/3
8. Sequencing<br>*Describe how you would make a cup of tea.* Response: .... /1<br>A correct answer contains two or more appropriate stages in the right order.	/1
9. Definitions<br>*Describe what the following words mean:*<br>a. home Response: .... /1<br>b. search Response: .... /1<br>c. ambitious Response: .... /1	/3
10. Verbal reasoning<br>*I'd like you to tell me:*<br>a. why you would use an umbrella Response: .... /1<br>b. why people go on holiday Response: .... /1<br>c. what would you do if you were locked out of the house Response: .... /1	/3
**Total expressive skills score**	**/11**
☐ Below normal ☐ ≥ Normal (age 59 and under ≥17; age 60–69 ≥16; age 70 and over ≥15) **TOTAL SCORE**	**/20**

Overall impression, communication is:
☐ Functional and no changes identified
☐ Functional but deficits observed or reported, SP review indicated
☐ Not functional or restricted communication, URGENT SP review indicated

☐ **Refer to SP for communication** (reports changes; observed deficits in informal communication screen; stop and refer prompt ticked; below norm or unable to complete Sheffield)

Swallowing

Dysphagia screen completed by: ☐ Nursing ☐ SP ☐ Neither Outcome: ☐ Pass ☐ Fail
Current diet/fluids: ....
Have you noticed any difficulties with your swallowing? ☐ Yes ☐ No If YES, details: ....
Drooling or pooling secretions observed? ☐ Yes ☐ No If YES, details: ....

☐ **Refer to SP for swallowing**

Vision and Perception

Glasses: ☐ Yes ☐ No
Blurry vision: ☐ Yes ☐ No
Diplopia: ☐ Yes ☐ No
Nystagmus: ☐ Yes ☐ No
Reading: ☐ Intact ☐ Impaired

Visual tracking: ☐ Intact ☐ Impaired
Convergence: ☐ Intact ☐ Impaired
Visual field: ☐ Intact ☐ Impaired *(indicate →)*
Visuospatial neglect: ☐ Left ☐ Right ☐ No

L R

Comments: ....

☐ **Refer to OT for:** ....

Respiratory Assessment

Medical history: ☐ Asthma ☐ COPD ☐ Bronchiectasis ☐ Active lung cancer ☐ Other *(specify)*: ....
Shortness of breath: ☐ Yes ☐ No
Phlegm/mucus: ☐ Yes ☐ No
RR 9–20 bpm: ....
Difficulty breathing: ☐ Yes ☐ No
Cough on command: ☐ Strong ☐ Weak
SpO_2 >95% (>85% for COPD): ....

Safe to proceed to mobility assessment? ☐ Yes ☐ No
If NO, reason: ☐ Shortness of breath ☐ Difficulty breathing ☐ SpO2 ≤95% (≤85% for COPD)

☐ **Refer to PT for review** (medical history; "yes" to any question; weak cough; RR or SpO_2 outside range)

© Copyright Mater Misericordiae Limited. All rights reserved.

mater health

TRANSDISCIPLINARY INITIAL NEUROLOGICAL SCREENING ASSESSMENT (TINSA)

Unit Record No. ______________________

Surname ______________________

Given Names ______________________

DOB ____________ Sex ____________

AFFIX PATIENT IDENTIFICATION LABEL HERE

Upper and Lower Limbs

Dominance: ☐ Left ☐ Right Affected limb(s): ....

Hypertonicity: ☐ Left ☐ Right ☐ No ....

Oedema: ☐ Yes ☐ No .... Pain (VAS): ....

Limb	ROM/MMT		Light touch sensation		Proprioception	
	Left	Right	Left	Right	Left	Right
Upper limb						
Lower limb						

Coordination

FNF: ☐ Intact ☐ Impaired – details: ....

Diadochokinesis: ☐ Intact ☐ Impaired – details: ....

Heel-shin slide*: ☐ Intact ☐ Impaired – details: **Do not complete if poor skin integrity* ....

Foot-tapping: ☐ Intact ☐ Impaired – details: ....

Sensorimotor neglect: ☐ No ☐ Left ☐ Right

Shoulder precautions required? ☐ Yes ☐ No

If YES: ☐ Left ☐ Right ☐ "Support my arm" band provided ☐ Collar and Cuff provided ☐ Positioning charts provided

Comments: ....

....

....

....

Safe to proceed to mobility assessment? ☐ Yes ☐ No If NO, reason: ☐ Cognition ☐ Neglect ☐ LL < 3/5 ☐ LL ataxia

Refer to ☐ OT ☐ PT for: ....

Functional Mobility Assessment

Vestibular screen Vestibular Screening Tool (VST): .... /8 **Stop and refer to PT if VST ≥4/8 (do not complete mobility assessment)*

Vital signs SBP (mmHg): .... HR (BMP): .... Temparture (°C): ....

Check observation chart for usual SBP if: ☐ SBP <110mmHg ☐ SBP >160mmHg

Medical clearance if: ☐ SBP <90mmHg ☐ SBP >180mmHg ☐ HR <40bmp ☐ HR >120bpm

Stop and refer to PT if: ☐ SBP drops ≥20mmHg with change of position ☐ Fevers/>38.5°C

Current LOF

Bed mobility: ☐ Independent ☐ SBA ☐ Assist **Stop and refer to PT if "assist"* ....

Sitting balance: ☐ Independent ☐ SBA ☐ Assist **Stop and refer to PT if "SBA"/"assist"* ....

Transfers: ☐ Independent ☐ SBA ☐ Assist **Stop and refer to PT if "assist"/new aid* ....

Standing balance: ☐ Independent ☐ SBA ☐ Assist **Stop and refer to PT if "assist"/new aid* ....

Mobility: ☐ Independent ☐ SBA ☐ Assist **Stop and refer to PT if "assist"/new aid* ....

10 meter walk test (10MWT) Steps: .... Time: ...... : ...... Step length: .... Velocity: ....

Timed up and go (TUG) Time: ...... : ...... Mobility aid: ....

☐ **Refer to PT** **Below normal:** ☐ **10MWT** ☐ **TUG**

Binding margin - do not write. Do not reproduce by photocopying.
All clinical form creation and amendments must be conducted through Health Information.

© Copyright Mater Misericordiae Limited. All rights reserved.

mater health

TRANSDISCIPLINARY INITIAL NEUROLOGICAL SCREENING ASSESSMENT (TINSA)

Unit Record No. ____________________

Surname ____________________

Given Names ____________________

DOB ____________ Sex ____________

AFFIX PATIENT IDENTIFICATION LABEL HERE

Mood

I'm going to show you some words and pictures, can you pick the one(s) that describes or looks like how you are feeling?

☐ Angry ☐ Scared ☐ Worried ☐ Overwhelmed ☐ Sad ☐ Alone ☐ Vulnerable

☐ Neutral ☐ Calm ☐ Safe ☐ Positive ☐ Happy ☐ Hopeful

Do you have any stressors or worries while you stay in hospital? ☐ Yes ☐ No

If YES, details: ....

Affect observations: ☐ Elated ☐ Teary ☐ Flat/low mood ☐ Anxious ☐ Restless/agitated/fidgety ☐ Avoids eye contact ☐ Dismissive/passive

☐ Other *(specify)*: ....

☐ **Refer to SW** (e.g. anxiety/depression, psychosocial stressors, clinician concerns, incongruent self-report vs. affect observations)

Other observations (e.g. fatigue, patient concerns)

....

Summary of Assessment and Plan

Allied Health clinical impressions:

....

Referrals

☐ **OT:** Cognitive assessment (if confirmed TIA/stroke) ....

☐ **PT:** ....

☐ **SP:** ....

☐ **SW:** ....

☐ **Other:** ....

Rehabilitation candidate: ☐ Yes ☐ No ☐ Further assessment required

Safe to discharge: ☐ Yes ☐ No

Name:	Designation:	Signature:	Date:	Time (24hr):

Binding margin - do not write. Do not reproduce by photocopying.
All clinical form creation and amendments must be conducted through Health Information.

© Copyright Mater Misericordiae Limited. All rights reserved.
