# Supplementary material for: Transdisciplinary allied health assessment for patients with stroke: a pre-/post- mixed methods study protocol
Source: BMC Health Serv Res. 2022 Dec 24;22:1578. doi: 10.1186/s12913-022-08926-y (PMC9789550; doi:10.1186/s12913-022-08926-y)
Supplement: Supplementary file 5 — Additional file 5. Patient Satisfaction Survey. Description. Data collection form used to obtain patient satisfaction data. [file 12913_2022_8926_MOESM5_ESM.pdf]

Patient Satisfaction Survey

1. Were you seen by:

☐ Occupational Therapy ☐ Physiotherapy ☐ Speech Pathology ☐ Other

☐ Social Work ☐ Nursing ☐ Dietetics ☐ I don't know who was who

2. You would have been asked lots of questions about your living arrangements and people who support you, your home set-up, your vision, about your arms, legs, speech and thinking. Were you asked the same questions repeatedly?

☐ Yes (including the response "sometimes") ☐ No (including the response: "I don't think so")

2a. If yes, were you asked many times about:

☐ Living arrangements ☐ People who support you ☐ Previous walking ability

☐ Previous ability to complete daily activities ☐ Home set-up ☐ Arms

☐ Legs ☐ Vision ☐ Thinking

☐ Speech/Communication ☐ Swallow ☐ Other ______________

3. Do you feel that the staff who asked you these questions completed a thorough assessment and understood your circumstances?

☐ Yes (including the response "I think so") ☐ No (including the response "I'm not sure")

4. Any comments (about your experience with the assessment/s)?

____

____

____

5. You have been admitted to hospital following a health event. In terms of your healthcare, what is important to you following this event (e.g., being assessed by certain professionals, access to therapy, discharge home as soon as possible, being prescribed the right medication, other aspects or goals of care)?

____

____

____

____
