# Supplementary material for: Transdisciplinary allied health assessment for patients with stroke: a pre-/post- mixed methods study protocol
Source: BMC Health Serv Res. 2022 Dec 24;22:1578. doi: 10.1186/s12913-022-08926-y (PMC9789550; doi:10.1186/s12913-022-08926-y)
Supplement: Supplementary file 6 — Additional file 6. Staff Satisfaction Survey. Description. Data collection form used to obtain staff satisfaction data. [file 12913_2022_8926_MOESM6_ESM.pdf]

Staff Satisfaction Survey

1. What is your role on the ward?

☐ Allied Health ☐ Nurse ☐ Doctor ☐ Other_____________

2. How many years have you worked in stroke care?

☐ Less than 5 years ☐ 5 – 10 years ☐ 10 – 15 years ☐ More than 15 years

3. Did the Transdisciplinary Initial Neurological Screen help you to be more efficient or less efficient in your practice?

☐ More efficient ☐ Less efficient ☐ No change ☐ Don't know what that is

4. What were the benefits of the Transdisciplinary Initial Neurological Screen?

☐ Reduced time spent completing assessment

☐ Reduced time spent reading allied health assessment

☐ Helped to efficiently formulate my plan

☐ Allied health assessments are located in the same place

☐ Helped to complete Nursing care pathway

☐ Patients weren't repeatedly asked the same questions

☐ Improved collaborative team approach

☐ Helped me make appropriate allied health referrals

☐ I didn't notice any change

☐ Other ____

5. Do you feel confident that the information on the Transdisciplinary Initial Neurological Screen is reliable and accurate?

☐ Yes ☐ No ☐ Unsure

5a. And what makes you feel this way?

____

____

6. What were the challenges of using the Transdisciplinary Initial Neurological Screen? And are there any changes that could be made that would assist your practice?

____

____

____

7. Compared to the usual assessment process (i.e. discipline-specific assessments), do you think anything was overlooked or foregone by utilising the novel TINS?

8. Any other comments?
