# Supplementary material for: Transdisciplinary allied health assessment for patients with stroke: a pre-/post- mixed methods study protocol
Source: BMC Health Serv Res. 2022 Dec 24;22:1578. doi: 10.1186/s12913-022-08926-y (PMC9789550; doi:10.1186/s12913-022-08926-y)
Supplement: Supplementary file 7 — Additional file 7. Interprofessional Staff Trust Survey. Description. The fit-for-purpose data collection form (i.e., not the validated team trust survey) used to obtain interprofessional staff trust data. [file 12913_2022_8926_MOESM7_ESM.pdf]

Interprofessional Staff Trust Survey

INSTRUCTION:

In all of the following questions, we ask you to consider a scenario in which a health practitioner (from another profession/discipline) is **assessing** your patient, and where the **findings of that assessment will directly impact** on how you will treat that patient when you next see the patient.

1. What professional group do you belong to?

- ☐ Medical
- ☐ Nursing
- ☐ Allied Health and Assistants
- ☐ Other

2. How much would you trust another health professional to look out for the patient's interests?

0_______1_______2_______3_______4_______5_______6_______7_______8_______9______10

0 – not at all 10 - completely

3. How much would you trust another health professional to be open and discuss their assessment findings with you?

0_______1_______2_______3_______4_______5_______6_______7_______8_______9______10

0 – not at all 10 - completely

4. How much would you trust another health professional to tell you the truth about their findings?

0_______1_______2_______3_______4_______5_______6_______7_______8_______9______10

0 – not at all 10 - completely

5. How much would you trust another health professional to do what needs to be done (e.g. complete an assessment) without errors?

0_______1_______2_______3_______4_______5_______6_______7_______8_______9______10

0 – not at all 10 - completely

Remember: we ask you to consider a scenario in which a health practitioner (from another profession/discipline) is **assessing** your patient, and where the **findings of that assessment will directly impact** on how you will treat that patient when you next see them.

6. How much would you trust another health professional to protect private patient information?

0_______1_______2_______3_______4_______5_______6_______7_______8_______9______10

0 – not at all 10 - completely

7. How much would you trust another health professional to do what is best for the patient?

0_______1_______2_______3_______4_______5_______6_______7_______8_______9______10

0 – not at all 10 - completely

8. How much would you trust another health professional to reliably do an assessment that has implications for what you will do when you next see the patient?

0_______1_______2_______3_______4_______5_______6_______7_______8_______9______10

0 – not at all 10 - completely

9. How much would you trust another health professional to see the patient in a timely way when they are admitted to the ward?

0_______1_______2_______3_______4_______5_______6_______7_______8_______9______10

0 – not at all 10 - completely

Still thinking about a scenario in which a health practitioner (from another profession/discipline) is **assessing** your patient, and where the **assessment findings will impact** on how you will treat that patient when you next see them:

10. Thinking about a context in which health practitioners work across disciplines (e.g., nurses assessing some tasks that doctors might usually perform), how much would you trust the Mater to support such a model of health service delivery?

0_______1_______2_______3_______4_______5_______6_______7_______8_______9______10

0 – not at all 10 - completely

11. Thinking about a context in which health practitioners work across disciplines (e.g., nurses assessing some tasks that doctors might usually perform), would you agree to such a model of care/assessment at the Mater?

0_______1_______2_______3_______4_______5_______6_______7_______8_______9______10

0 – not at all 10 - completely

12. Do you believe someone who is also trained in the transdisciplinary assessment (known as the multidisciplinary assessment on the AMU) would arrive at the same result as you?

☐ Yes ☐ No ☐ Unsure

Can you explain why you chose the above answer?

____

____

____

A final open-ended opportunity for comment:

Please provide us with your thoughts about the idea of, or potential for, transdisciplinary care/assessment at the Mater:

____

____

____

____
