# Supplementary material for: Transdisciplinary allied health assessment for patients with stroke: a pre-/post- mixed methods study protocol
Source: BMC Health Serv Res. 2022 Dec 24;22:1578. doi: 10.1186/s12913-022-08926-y (PMC9789550; doi:10.1186/s12913-022-08926-y)
Supplement: Supplementary file 8 — Additional file 8. Staff Confidence Survey. Description. Data collection form used to obtain staff confidence data over time. [file 12913_2022_8926_MOESM8_ESM.pdf]

Building Staff Confidence Survey

1. Please indicate your profession:

- ❑ Occupational therapist (go to Q2, and answer all questions up to and including Q7)
- ❑ Physiotherapist (go to Q2, and answer all questions up to and including Q7)
- ❑ Speech Pathologist (go to Q8, and answer all questions up to and including Q11)
- ❑ Social Worker (go to Q12, and answer all questions up to and including Q15)

*If you are an **occupational therapist** or **physiotherapist,** please answer questions 2 – 7. Otherwise, please skip to question 8.*

2. How many years of experience do you have working as an occupational therapist or physiotherapist?

__________ years

3. Did you help develop the module content with the Transdisciplinary Initial Neurological Screening Assessment (TINSA) project working party?

- ❑ Yes
- ❑ No

4. How long have you been administering the TINSA?

- ❑ 1 month or less
- ❑ 1 - 2 months
- ❑ 2 – 3 months
- ❑ 3 – 4 months
- ❑ 4 – 5 months
- ❑ 5 – 6 months
- ❑ 6 months or more

5. How many times have you administered the TINSA?

- ❑ 5 or less
- ❑ 6 – 10
- ❑ 11 – 14
- ❑ 15 or more

Remember that your rating of confidence does not necessarily reflect your clinical skills, so please answer the following questions as honestly as you can.

6. Right now, on a scale of 1 (no confidence) to 10 (high confidence) how would you rate your confidence in completing the TINSA with patients?

|________|________|________|________|________|________|________|________|________|

1 2 3 4 5 6 7 8 9 10

7. Right now, what assessments tasks or questions are you NOT confident with? (Select all that apply).

- ❑ Social History
- ❑ Mood
- ❑ Sheffield Screening Test for Acquired Language Disorders
- ❑ Respiratory assessment
- ❑ Visual screens
- ❑ Perceptual screens
- ❑ Upper limb assessments
- ❑ Lower limb assessments
- ❑ Shoulder precautions
- ❑ Functional mobility assessment
- ❑ 10-meter walk test
- ❑ Timed Up and Go (TUG)
- ❑ Other tasks or questions (please list):

____

____

____

*If you are an **occupational therapist** or **physiotherapist**, this is the end of the survey. Thank you for your participation.*

If you are a ***speech pathologist,*** *please answer questions 8 - 11. Otherwise, please skip to question 12.*

Remember that your rating of confidence does not necessarily reflect the clinical skills of your colleagues, so please answer the following questions as honestly as you can.

8. Right now, on a scale of 1 (no confidence) to 10 (high confidence) how would you rate your confidence in the <u>occupational therapists</u> to complete the <u>communication section</u> of the TINSA?

|________|________|________|________|________|________|________|________|________|

1 2 3 4 5 6 7 8 9 10

9. Right now, on a scale of 1 (no confidence) to 10 (high confidence) how would you rate your confidence in the <u>occupational therapists</u> to <u>identify appropriate referrals</u> to speech pathology?

|________|________|________|________|________|________|________|________|________|

1 2 3 4 5 6 7 8 9 10

10. Right now, on a scale of 1 (no confidence) to 10 (high confidence) how would you rate your confidence in the <u>physiotherapists</u> to complete the <u>communication section</u> of the TINSA?

|________|________|________|________|________|________|________|________|________|

1 2 3 4 5 6 7 8 9 10

11. Right now, on a scale of 1 (no confidence) to 10 (high confidence) how would you rate your confidence in the <u>physiotherapists</u> to <u>identify appropriate referrals</u> to speech pathology?

|________|________|________|________|________|________|________|________|________|

1 2 3 4 5 6 7 8 9 10

If you are a ***speech pathologist****, this is the end of the survey. Thank you for your participation.*

*If you are a **social worker,** please answer questions 12 -15. Otherwise, you have completed the survey, thank you for your participation.*

Remember that your rating of confidence does not necessarily reflect the clinical skills of your colleagues, so please answer the following questions as honestly as you can.

12. Right now, on a scale of 1 (no confidence) to 10 (high confidence) how would you rate your confidence in the <u>occupational therapists</u> to complete the <u>social history and mood section</u> of the TINSA?

|________|________|________|________|________|________|________|________|________|

1 2 3 4 5 6 7 8 9 10

13. Right now, on a scale of 1 (no confidence) to 10 (high confidence) how would you rate your confidence in the <u>occupational therapists</u> to <u>identify appropriate referrals</u> to social work?

|________|________|________|________|________|________|________|________|________|

1 2 3 4 5 6 7 8 9 10

14. Right now, on a scale of 1 (no confidence) to 10 (high confidence) how would you rate your confidence in the <u>physiotherapists</u> to complete the <u>social history and mood section</u> of the TINSA?

|________|________|________|________|________|________|________|________|________|

1 2 3 4 5 6 7 8 9 10

15. Right now, on a scale of 1 (no confidence) to 10 (high confidence) how would you rate your confidence in the <u>physiotherapists</u> to <u>identify appropriate referrals</u> to social work?

|________|________|________|________|________|________|________|________|________|

1 2 3 4 5 6 7 8 9 10

*If you are a **social worker**, this is the end of the survey. Thank you for your participation.*
